# Pain-Associated Transcriptome Changes in Synovium of Knee Osteoarthritis Patients

**DOI:** 10.3390/genes9070338

**Published:** 2018-07-04

**Authors:** Anna Bratus-Neuenschwander, Francesc Castro-Giner, Mojca Frank-Bertoncelj, Sirisha Aluri, Sandro F. Fucentese, Ralph Schlapbach, Haiko Sprott

**Affiliations:** 1 Functional Genomics Center Zurich, University of Zurich/ETH Zurich, 8057 Zurich, Switzerland; francesc.castro@unibas.ch (F.C.-G.); sirisha.aluri@fgcz.ethz.ch (S.A.); ralph.schlapbach@fgcz.ethz.ch (R.S.); 2 Center of Experimental Rheumatology, Department of Rheumatology, University Hospital Zurich/University of Zurich, BioTechno Park Schlieren, 8952 Schlieren, Switzerland; Mojca.Frank@usz.ch; 3 Department of Orthopaedic Surgery, Balgrist University Hospital, University of Zurich, 8008 Zurich, Switzerland; Sandro.Fucentese@balgrist.ch; 4 Arztpraxis Hottingen, 8032 Zurich, Switzerland; prof.haiko.sprott@hin.ch; 5 Medical Faculty, University of Zurich, 8091 Zurich, Switzerland

**Keywords:** pain, osteoarthritis, knee, synovium, RNA-seq

## Abstract

Joint pain causes significant morbidity in osteoarthritis (OA). The aetiology of joint pain in OA is not well understood. The synovial membrane as an innervated joint structure represents a potential source of peripheral pain in OA. Here we analyse, using a hypothesis-free next generation RNA sequencing, the differences in protein-coding and non-coding transcriptomes in knee synovial tissues from OA patients with high knee pain (*n* = 5) compared with OA patients with low knee pain (*n* = 5), as evaluated by visual analogue scale (VAS). We conduct Gene Ontology and pathway analyses on differentially expressed mRNA genes. We identify new protein-coding, long non-coding RNA and microRNA candidates that can be associated with OA joint pain. Top enriched genes in painful OA knees encode neuronal proteins that are known to promote neuronal survival under cellular stress or participate in calcium-dependent synaptic exocytosis and modulation of GABA(γ-aminobutyric acid)ergic activity. Our study uncovers transcriptome changes associated with pain in synovial microenvironment of OA knees. This sets a firm ground for future mechanistic studies and drug discovery to alleviate joint pain in OA.

## 1. Introduction

Primary knee osteoarthritis (OA) is the most common joint disorder, affecting more than 10% of the Western European population aged 60 years or older [1]. Joint pain and functional disability decrease quality of life in OA patients [2]. OA affects hands, feet, knees, hips and spine [3] with structural damage in the articular cartilage, subchondral bone, ligaments, tendons, menisci, muscles, synovium and nerve tissues [4]. The ethiopathogenesis of joint pain in OA is not well understood [5] and the therapeutic strategies to relieve OA pain are therefore limited. The correlation between the severity of pain and the degree of radiographic changes in OA knees is poor [6,7].

A number of studies substantiate the role of the central nervous system in the ethiopathogenesis of chronic pain in OA [8,9], nevertheless the sources of peripheral nociception are increasingly explored [6,10]. Peripheral pain can originate in any of the innervated joint structures, including the synovium [5]. Synovial inflammation in OA causes joint pain [11]. Inflammation decreases the activation threshold of local afferent nerve fibers in response to mechanical stimuli resulting in peripheral sensitisation. Additionally, interactions between a damaged joint and the sensory nervous system cause pain [11]. Most likely, there are two interconnected mechanisms with reverse causality: Joint damage causes synovial inflammation, whereas neurogenic inflammation contributes to joint damage, creating a positive feedback loop [12].

Genes involved in pain pathways in the nervous system are also expressed in non-neuronal cells of the joint [13]. Epidemiology studies identified a panel of candidate genes and functional genetic variants associated with OA pain [14]. To date, no hypothesis-free study with a genome-wide approach has been conducted to interrogate the molecular basis of OA pain.

Here we analyse by RNA sequencing the transcriptomes of knee synovial tissues from OA patients with high and low intensity knee pain as assessed by visual analogue scale (VAS). We uncover the molecular complexity of OA joint pain and reveal novel candidate genes associated with knee OA pain.

## 2. Materials and Methods

### 2.1. Patient Information

OA patients (Table 1) were enrolled in the study at the Uniklinik Balgrist, Zurich, Switzerland. Pain was assessed a day before surgery by a clinical investigator using a 10-point VAS. Patients with low (VAS scores: 0–3) or high (VAS 7–10) pain were enrolled. The indication for surgery in patients with low pain were a relevant deformity of the axis, mainly valgus, and/or loss of bone stock with a symptomatic instability and risk to fall. The Local Ethical Committee (KEK-ZH-Nr. 2013-0210) approved the study. All patients gave written informed consent. All experiments were performed in accordance with relevant guidelines and regulations.

### 2.2. Sample Collection and Total RNA Extraction

The standardized surgical excision (excision of the capsule with synovia in the recessus suprapatellaris) was performed. The biopsies (two per patient with the size of approximately 2 cm^2^) were fresh frozen (dry ice) and RNA was extracted randomly from one biopsy using TRIzol^®^ Reagent (Thermo Fisher Scientific, Waltham, MA, USA). RNA was analysed by Qubit^®^ 1.0 Fluorometer (Thermo Fisher Scientific) and Agilent 2200 TapeStation (Agilent Technologies, Santa Clara, CA, USA); RNA with RIN (RNA Integrity Number) values 8.3–9.1 was used for sequencing.

### 2.3. Next Generation Sequencing

Libraries for mRNA/long noncoding RNA (lncRNA) and small RNA sequencing were prepared using the Illumina (San Diego, CA, USA) TruSeq^®^ Stranded total RNA Sample Preparation Kit and the NEBNext^®^ Small RNA Library Prep Set for Illumina^®^, respectively. Libraries were quantified and quality checked using quantitative polymerase chain reaction (qPCR) (Roche, Basel, Switzerland) with Illumina adapter specific primers and Agilent 2200 TapeStation, respectively. Diluted indexed mRNA-seq (10 nM) and small RNA-seq (1 nM) libraries were pooled, used for cluster generation (Illumina TruSeq SR Cluster Kit v4-cBot-HS) and sequenced (Illumina HiSeq 2500, Illumina TruSeq SBS Kit v4-HS reagents, single read approach (1 × 125 bp) with 20–30 million reads per sample for mRNA/lncRNA, 10–20 million reads for miRNA).

### 2.4. Data Analysis

After quality control (FastQC, Babraham Bioinformatics, https://www.bioinformatics.babraham.ac.uk/) we quantified transcript expressions using the RSEM package (version 1.2.18) [16]. Transcript references were built using the Ensembl version 75 annotations of the GRCh37.p13 human genome assembly. The expression level for each gene was obtained by the aggregation of counts from all gene isoforms. Genes with less than 10 counts in ≥50% of samples for each condition were excluded from the analysis. Small RNA reads were processed using the ncPRO-seq [17] and annotated using miRbase version 20 [18], RepeatMasker and Rfam [19].

Differentially expressed genes were identified using the R/bioconductor package edgeR (v3.14.0) [20]. The Benjamini-Hochberg procedure [21] was used to adjust *p* values and genes with an adjusted *p* value ≤ 0.11 were considered differentially expressed. Gene ontology (GO) enrichment analysis was conducted using the Overrepresentation Test from PANTHER web portal (release 2015043074) [22] on biological process ontologies, using Fisher’s exact test option and all genes in the database as a background. Redundant GO terms were removed using the REVIGO portal [23]. Pathway analysis was performed using the gene set enrichment analysis tool using MSigDB curated gene sets (C2, release 5) [24,25]. GO terms and pathways with adjusted *p* value ≤0.05 were considered enriched. Small RNAs with an absolute log2 fold change ≥1 and *p* value ≤0.05 were defined as differentially expressed.

Deconvolution of synovial tissue into immune cell types was evaluated using an mRNA machine learning approach of CIBERSORT [26], running the relative and absolute modes and using 500 permutations. Input matrix contained fragments per kilobase million (FPKM) for all expressed genes. CIBERSORT uses the reference dataset LM22 that consist of 22 distinct immune cell types and was constructed from gene expression profiles of those cell types measured on the Affymetrix U133A/Plus2 (Santa Clara, CA, United States and Illumina Expression BeadChip (HumanHT-12 v4) platforms.

The raw and processed RNA-seq data have been deposited under Gene Expression Omnibus (GEO) accession number GSE99662.

## 3. Results

### 3.1. Protein-Coding Transcriptome and Gene Enrichment Analysis

We identified 32 genes that were differentially expressed in the synovium of OA patients with low and high pain (Table 2). Among these 32 mRNAs, 30 mRNAs were up-regulated in OA synovium from patients with high pain compared with patients experiencing low pain. The top up-regulated genes were the Stress Responsive DNAJB4 Interacting Membrane Protein (*SDIM1*), Carboxypeptidase E (*CPE*) and Otoferlin (*OTOF*). Ankyrin Repeat Domain 20 Family Member A4 (*ANKRD20A4*) and pentraxin 3 (*PTX3*) were the only down-regulated genes in OA synovium from patients experiencing high pain (Table 2).

To identify biological processes enriched in the synovium of OA patients with high knee pain, we performed the GO enrichment analysis and gene set enrichment analysis (GSEA) on the 32 differentially expressed genes. Six GO sets were enriched in patients with high pain (Table 3). Only two of the enriched GO term have a relative small size (<200 genes), Peripheral nervous system development (GO:0007422) and Second-messenger-mediated signalling (GO:00019932). The characteristics of the 18 genes that were enriched in these GO terms are described in the Table 4 and Table S1. We identified two enriched Reactome pathways, specifically Nerve growth factor (NGF) signalling via Tropomyosin receptor kinase A (TRKA) from the plasma membrane and Signalling by NGF (Table 5). These two pathways were redundant with four significantly enriched genes including adenylate cyclase 3 (*ADCY3*), Src homology 2 domain containing (*SHC*) transforming protein 2 (*SHC2*), neurotrophic receptor tyrosine kinase 2 (*NTRK2*) and dual specificity phosphatase 4 (*DUSP4*).

### 3.2. Noncoding Transcriptome

Two lncRNAs (RN7SL3 and RP11-195E2.1) and thirty-five miRNAs were identified as differentially expressed in the synovium of OA patients with contrast pain phenotypes (Table 1 and Table 6). Twenty-one miRNAs were down-and fourteen were up-regulated in patients with high compared with low knee pain, but these differences were not significant when correcting for false discovery rate (FDR) (Table 6).

### 3.3. Deconvolution Analysis of Harvested Synovial Tissue

**Figure 1:**
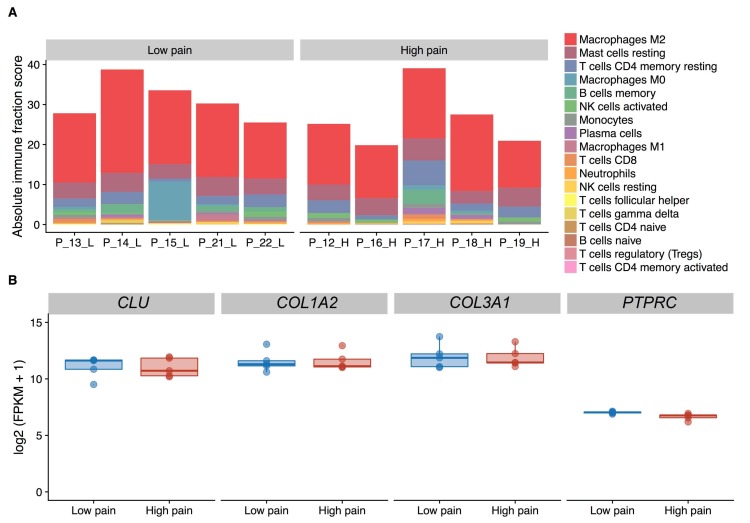
Deconvolution analysis of the cell composition in synovial tissue extracts based on RNA-sequencing data. (**A**) Fraction of leukocyte type content as inferred by CIBERSORT. (**B**) Expression levels of fibroblast (*CLU*, *COL1A2*, *COL3A1*) and leukocyte (*PTPRC*) marker genes. FPKM, Fragments Per Kilobase Million.

There were no major differences in immune cell composition (Kruskal-Wallis test, adjusted *p* value ≤ 0.05) nor in the absolute content of immune cells (Kruskal-Wallis test, *p* = 0.17) in the synovium between patients (Figure 1A). The expression level of *CD45* (*PTPRC*), a leukocyte marker (Figure 1B) did not differ between synovial tissues from patients with high and low pain (adjusted *p* = 0.78). In addition, fibroblast markers *CLU*, *COL1A2*, *COL3A1*, identifying for different subpopulations of synovial fibroblasts [27] were expressed at high levels in synovial tissues (log2 FPKM > 9) and their expression did not differ between patients with contrast pain phenotypes (Figure 1B).

## 4. Discussion

Pain is a subjective and complex sensory experience and evaluation of pain is rather difficult. Among several methods for pain assessment [28], we used VAS since it is a clinical routine, well accepted in scientific pain literature and it influences functional scores [29]. Patients with contrast pain phenotypes were matched for gender (females), smoking status (non-smokers) and ethnic origin (Europeans) which can confound experiencing the pain [6]. Additionally, patient groups with contrast pain phenotypes did not differ significantly in age (70.6 ± 6.5 vs. 71.0 ± 6.0 in high vs. low pain group), Body Mass Index (BMI) (31.1 ± 8.5 vs. 29.0 ± 5.1 in high vs. low pain group) or the extent of joint damage (3 and 4 in Kellgren and Lawrence grade system). The use of analgesics could decrease pain in the low pain group patients 021 and 022, but appeared inefficient in controlling the pain in the high pain group patients 016, 018, 019. 

We uncovered a signature of genes that are differentially expressed in the synovial tissues from knees of OA patients with contrast pain phenotypes. The synovial inflammatory mediators that are known to induce and/or respond to pain [12] were not enriched in the transcriptome profiles in synovial tissues from patients with high compared to patients with low knee pain. The deconvolution analysis of RNA-seq data showed no differences in the composition of immune cells or synovial fibroblasts between patients. Instead, the three top differentially expressed genes in patients with high pain are neuronal proteins. Two of them, specifically *SDIM1* and *CPE*, are known for their roles in cellular responses to stress. This suggests a potential molecular connection between chronic pain and stress, as also recently demonstrated by Descalzi et al. [30].

SDIM1 is involved in cellular response to stress and inhibition of cell death [31]. In NT2 neurons SDIM1 exhibits a bi-phasic response to cell death-inducing injuries; initial down-regulation of SDIM1 is followed by an up-regulation in surviving cells [31]. Overexpression of *SDIM1* improves survival of neuro-progenitor cells after injury, substantiating the pro-survival effects of SDIM1 under stress conditions [31]. We show an increased expression of *SDIM1* in knee synovial tissues from OA patients with high pain. Our data suggest that acting as a stressor, the chronic pain might increase the expression of *SDIM1* in the synovial microenvironment of OA knees to activate its protective functions. 

Besides *SDIM1*, *CPE* can steer cellular responses to stress and we show that also CPE is significantly up-regulated in the synovium of OA patients with high pain. CPE acts as a trophic factor and promotes neuronal survival upon various stressors via increasing anti-apoptotic protein BCL-2 Bcl-2 in a MEK/ERK and/or PI3-K/AKT-dependent manner [32]. Stress-driven increase of CPE in brain has pro-survival effects in neurons [32]. Considering the sensitivity of CPE in responding to different stressors, pain as a stressor might induce CPE expression in OA knees. CPE acts through a similar molecular mechanism like brain derived neurotrophic factor (BDNF) [33] which is expressed in knee synovium of OA patients [13], its expression however does not differ between OA patients with contrast pain phenotypes. This suggests that CPE and BDNF may not function together to promote cell survival in painful OA joints; a similar scenario proposed for hippocampal neurons [32]. Instead, we show increased expression of *IGF1*, which is known to have neuroprotective and pro-survival effects by activating ERK and Akt [34].

Collectively, these results suggest that the cell protective functions of SDIM1 and CPE might be needed within the stress microenvironment of the OA synovium in patients with high pain. CPE and SDIM1 appear striking molecular candidates for future studies due to its potential joint protective effects.

*OTOF* was the second of the top three up-regulated genes in the synovium of OA patients with high pain. OTOF is a transmembrane protein required for calcium-dependent synaptic exocytosis in cochlear sensory cells [35] and mutations in OTOF cause deafness [35]. Besides, OTOF can modulate the GABAergic activity in the GAD65-dependent manner in neuronal and non-neuronal cells [36]. Future studies might reveal whether OTOF can change the secretion of GABA in the OA synovium in response to pain, thereby modulating the pain transmission. 

In the gene networks enrichment analysis using GO terms and Reactome pathways, the networks that include *SDIM1* and *OTOF* did not appear enriched, possibly due to a rather limited published data on the function of these genes. Meanwhile, CPE was linked to the anatomical structure morphogenesis. Several other up-regulated genes in the synovium of OA patients with high pain did not appear in enriched pathways and the knowledge about most of these genes is rather limited. Nevertheless, some of these genes participate in nervous system development (*CLSTN2*, *TUBB2B*) [37,38], inflammation including arthritis (*AOC3*) [39] and stress-induced responses (*C10orf10*) [40], making them promising molecular candidates in OA-driven pain.

On the other hand, the neurotrophic tyrosine kinase receptor type 2 (*NTRK2*), also known as TrkB was present in every enriched GO term and in both enriched Reactome pathways. This might suggest that NTRK2 is one of the key drivers, involved in the pain-related transcriptional changes in the OA synovium. The expression of the neurotrophic factors that function via TrkB including *BDNF*, nerve growth factor (*NGF*), was not altered in OA patients with high knee pain. This suggests that other molecular candidates might function via TrkB to activate downstream signaling pathways in the synovium of painful OA joint.

To date no studies have explored the global alterations in the synovial noncoding RNA expression in relation to OA joint pain. We identified two differentially expressed lncRNAs and 35 differentially expressed miRNAs in the synovium of OA patients with contrast pain phenotypes, but the differences in the miRNA expression were not significant when correcting for FDR. In all this suggests that pain-related transcriptional changes primarily affect the protein coding transcriptome. Pain-associated changes in miRNAs in OA appear less frequent or smaller, thus larger sample sizes might be required for miRNA studies.

The two identified lncRNAs (*RN7SL3* and *RP11-195E2.1*), which were strongly down-regulated in OA patients with high knee pain, have largely unknown function. In contrast, miR-146a-3p, the top differentially expressed miRNA in the synovium of OA patient with contrast pain phenotypes, has already been linked to the pain-related pathophysiology of knee OA [41]. MiR-146a-3p, which regulates the cell repair responses to tissue damage. It was also shown to be up-regulated in the cartilage and synovium of OA patients compared with healthy controls, which directly links this miRNA to OA pathogenesis [41]. We show that miR-146a-3p is down-regulated in the synovium of OA patients with high pain. This is in line with the observed down-regulation of miR-146a-3p in peripheral and central neurons in an animal model of OA-pain [41], making miR-146a-3p a prominent molecular candidate in pain-related pathology in OA.

## 5. Conclusions

In summary, the molecular dynamics in the synovium of painful OA joints indicate that the synovium is an important tissue to investigate in the discovery of novel therapeutics to decrease OA joint pain. The increased expression of stress-inducible molecules with protective roles in cell survival emerges as a common theme in OA patients with high intensity knee pain. Future studies of stress-induced protective mechanisms should further deepen the understanding of joint pain in OA.

## Figures and Tables

**Table 1:** Information about the patients enrolled into study.

Patient No.	Sex	Age	Nationality	Smoking Status	Drug Therapy	Classes of Medicaments	Recommended Procedure for Surgery	Degree of Radiographic Changes in the Knee ^3^	Current Laboratory Data: CRP ^4^/BSG ^5^	Earlier Surgery Procedures	BMI ^6^	VAS ^7^	Pain
012	F ^1^	76	Swiss	non	Pantozol, Pravastatin, Torasemid, Triatec	1, 5, 3, 4	TKA ^2^	4	8.3/18	Meniscus	27.5	8	high
013	F	64	Italian	non	Co-Aprovel	5	TKA	4	8.5/50	non	31.8	3	low
014	F	74	Swiss	non	non	non	TKA	4	1.2/20	Meniscus	23.4	0	low
015	F	75	Swiss	non	Lithium	6	TKA	4	0.5/12	non	23.7	1	low
016	F	62	Swiss	non	Metoprolol, Sirdalud, Femeston, Pantozol	2, 10, 7, 1	TKA	3	4.6/14	non	23.2	9	high
017	F	70	German	non	Fludex	3	TKA	4	-/-	non	43.7	8	high
018	F	78	Swiss	non	Atenolol, Co-Epril, Madopar, Metformin, Simcora, Tiatral, Zanidip, Tramadol	2, 4, 6, 8, 5, 10	TKA	4	7.7/22	non	35.5	7	high
019	F	67	Swiss	non	Tilur, Zolpidem	10, 6	TKA	4	1.1/7	non	25.4	8	high
021	F	65	Swiss	non	Aspirin, Surmontil	10, 6	TKA	4	5.4/17	Arthroscopy	32	0–3.5 ^8^	low
022	F	77	Italian	non	Aspirin, Calcimagon, Magnesium, Pantozol, Pemzek	10, 9, 1, 4	TKA	4	9.2/29	non	34.2	2	low

Classes of medicaments: (1) proton pump inhibitors, (2) β-blockers, (3) diuretics, (4) Angiotensin-converting enzyme (ACE) inhibitors, (5) drugs decreasing cholesterol blood level, (6) medicaments against psychiatric disorders, (7) hormonal replacement therapy, (8) diabetes, (9) drugs against osteoporosis, (10) pain killers; β-blockers, diuretics, ACE inhibitors and ZANIDIP (unclassified) are all against high blood pressure or heart disease. ^1^ F: female, ^2^ TKA: Total Knee Arthroplasty, ^3^ according to Kellgren and Lawrence system grade [15], ^4^ CRP: C–Reactive Protein, ^5^ BSG: Blood Sedimentation Rate, ^6^ BMI: Body Mass Index, ^7^ VAS: Visual Analog Scale, ^8^ patient with VAS at rest 0 and under loading with 3.5.

**Table 2:** List of genes and long noncoding RNAs differentially expressed in synovial tissue of patients with contrast pain phenotypes.

No.	Gene ID	Gene Name	Description	Type	log2 Fold Change	*p* Value	Adjusted *p* Value
1	ENSG00000256391	*SDIM1*	stress responsive DNAJB4 interacting membrane protein 1	protein_coding	2.913	2.83 × 10^−10^	4.66 × 10^−6^
2	ENSG00000115155	*OTOF*	otoferlin	protein_coding	3.42	7.70 × 10^−8^	0.000634
3	ENSG00000109472	*CPE*	carboxypeptidase E	protein_coding	1.107	2.13 × 10^−7^	0.001172
4	ENSG00000138944	*KIAA1644*	*KIAA1644*	protein_coding	1.184	1.93 × 10^−6^	0.007935
5	ENSG00000158258	*CLSTN2*	calsyntenin 2	protein_coding	1.214	6.48 × 10^−6^	0.01909
6	ENSG00000131471	*AOC3*	amine oxidase, copper containing 3	protein_coding	1.243	8.55 × 10^−6^	0.01909
7	ENSG00000141068	*KSR1*	kinase suppressor of ras 1	protein_coding	0.8648	9.15 × 10^−6^	0.01909
8	ENSG00000108551	*RASD1*	RAS, dexamethasone-induced 1	protein_coding	1.605	1.04 × 10^−5^	0.01909
9	ENSG00000042062	*FAM65C*	family with sequence similarity 65, member C	protein_coding	1.655	1.04 × 10^−5^	0.01909
10	ENSG00000138031	*ADCY3*	adenylate cyclase 3	protein_coding	0.8997	1.29 × 10^−5^	0.02012
11	ENSG00000120875	*DUSP4*	dual specificity phosphatase 4	protein_coding	1.297	1.42 × 10^−5^	0.02012
12	ENSG00000113448	*PDE4D*	phosphodiesterase 4D, cAMP-specific	protein_coding	1.083	1.47 × 10^−5^	0.02012
13	ENSG00000172014	*ANKRD20A4*	ankyrin repeat domain 20 family, member A4	protein_coding	−2.373	1.70 × 10^−5^	0.02151
14	ENSG00000221866	*PLXNA4*	plexin A4	protein_coding	1.232	2.43 × 10^−5^	0.02859
15	ENSG00000213494	*CCL14*	chemokine (C-C motif) ligand 14	protein_coding	1.212	3.10 × 10^−5^	0.03001
16	ENSG00000198947	*DMD*	dystrophin	protein_coding	0.9328	3.29 × 10^−5^	0.03006
17	ENSG00000166106	*ADAMTS15*	ADAM metallopeptidase with thrombospondin type 1 motif, 15	protein_coding	1.038	4.98 × 10^−5^	0.0432
18	ENSG00000135744	*AGT*	angiotensinogen (serpin peptidase inhibitor, clade A, member 8)	protein_coding	1.732	6.87 × 10^−5^	0.05489
19	ENSG00000129946	*SHC2*	SHC (Src homology 2 domain containing) transforming protein 2	protein_coding	1.574	7.00 × 10^−5^	0.05489
20	ENSG00000104332	*SFRP1*	secreted frizzled-related protein 1	protein_coding	0.953	7.41 × 10^−5^	0.05551
21	ENSG00000137285	*TUBB2B*	tubulin, beta 2B class IIb	protein_coding	1.592	8.44 × 10^−5^	0.06047
22	ENSG00000163431	*LMOD1*	leiomodin 1 (smooth muscle)	protein_coding	1.02	0.000143	0.09593
23	ENSG00000103196	*CRISPLD2*	cysteine-rich secretory protein LCCL domain containing 2	protein_coding	1.631	0.000146	0.09593
24	ENSG00000065320	*NTN1*	netrin 1	protein_coding	1.48	0.000154	0.09774
25	ENSG00000163661	*PTX3*	pentraxin 3, long	protein_coding	−2.075	0.000174	0.1009
26	ENSG00000144229	*THSD7B*	thrombospondin, type I, domain containing 7B	protein_coding	1.88	0.000181	0.1009
27	ENSG00000165507	*C10orf10*	chromosome 10 open reading frame 10	protein_coding	1.002	0.000185	0.1009
28	ENSG00000017427	*IGF1*	insulin-like growth factor 1 (somatomedin C)	protein_coding	1.159	0.000195	0.1009
29	ENSG00000148053	*NTRK2*	neurotrophic tyrosine kinase, receptor, type 2	protein_coding	1.176	0.000195	0.1009
30	ENSG00000162878	*PKDCC*	protein kinase domain containing, cytoplasmic	protein_coding	0.9649	0.000196	0.1009
31	ENSG00000171819	*ANGPTL7*	angiopoietin-like 7	protein_coding	1.825	0.000204	0.1017
32	ENSG00000111879	*FAM184A*	family with sequence similarity 184, member A	protein_coding	1.44	0.000219	0.1061
33	ENSG00000266037	*RN7SL3*	RNA, 7SL, cytoplasmic 3	other	−4.882	2.72 × 10^−5^	0.02982
34	ENSG00000230847	*RP11-195E2.1*		pseudogene	−2.975	2.90 × 10^−5^	0.02985

**Table 3:** Enriched Gene Ontologies terms for the 32 genes differentially expressed in synovial tissue of patients with contrast pain phenotypes.

Gene Ontology (GO) Term	Term ID	Ontology	Gene Set Size	Differentially Expressed Genes in Set	Expected Proportion	Fold Enrichment	Direction	Adjusted *p* Value
Peripheral nervous system development	GO:0007422	BP ^1^	74	4	0.11	>5	Up-regulation	3.62 × 10^−2^
Second-messenger-mediated signalling	GO:0019932	BP	149	5	0.22	>5	Up-regulation	2.13 × 10^−2^
Regulation of MAPK cascade	GO:0043408	BP	771	9	1.15	>5	Up-regulation	9.79 × 10^−3^
Regulation of protein phosphorylation	GO:0001932	BP	1330	11	1.98	>5	Up-regulation	1.44 × 10^−2^
Regulation of phosphorylation	GO:0042325	BP	1419	11	2.11	>5	Up-regulation	2.70 × 10^−2^
Anatomical structure morphogenesis	GO:0009653	BP	2341	14	3.49	4.02	Up-regulation	1.64 × 10^−2^

^1^ BP-biological processes.

**Table 4:** Gene Ontologies (GO) in the category of biological process and 18 enriched genes among 32 differentially expressed in synovial tissue of patients with contrast pain phenotypes. All enriched genes are up regulated in painful knee (Table 2).

Gene Ontology	*SDIM1*	*OTOF*	*CPE*	*KIAA1644*	*CLSTN2*	*AOC3*	*KSR1*	*RASD1*	*FAM65C*	*ADCY3*	*DUSP4*	*PDE4D*	*ANKRD20A4*	*PLXNA4*	*CCL14*	*DMD*	*ADAMTS15*	*AGT*	*SHC2*	*SFRP1*	*TUBB2B*	*LMOD1*	*CRISPLD2*	*NTN1*	*PTX3*	*THSD7B*	*C10orf10*	*IGF1*	*NTRK2*	*PKDCC*	*ANGPTL7*	*FAM184A*
Peripheral nervous system development														x		x		x											x			
Second-messenger-mediated signaling								x				x				x		x											x			
Regulation of MAPK cascade							x				x				x	x		x	x	x								x	x			
Regulation of protein phosphorylation							x			x	x	x			x	x		x	x	x								x	x			
Regulation of phosphorylation							x			x	x	x			x	x		x	x	x								x	x			
Anatomical structure morphogenesis			x				x				x			x		x		x	x	x		x	x	x				x	x	x		
Unclassified				x									x													x						x

**Table 5:** Results from the Gene Set Enrichment Analysis (GSEA) based on Reactome datasets.

Gene Set	Reactome ID	Description	Genes in Overlap	*p* Value	Adjusted *p* Value
NGF signaling via TRKA from the plasma membrane	R-HSA-187037	Genes involved in NGF signaling via TRKA from the plasma membrane	4	2.23 × 10^−6^	1.92 × 10^−3^
Signaling by NGF	R-HSA-166520	Genes involved in Signaling by NGF	4	1.38 × 10^−5^	5.92 × 10^−3^

**Table 6:** List of small RNAs significantly differentially expressed in synovial tissue of patients with contrast pain phenotypes; raw *p* value < 0.05 and absolute log2 fold change (logFC) ≥ 1; FDR: false discovery rate).

No.	Small RNA ID	logFC	*p* Value	FDR
1	hsa-miR-146a-3p	−3.0904	0.0021	0.9161
2	hsa-miR-3690	−2.0150	0.0061	0.9161
3	hsa-mir-6087	−1.0671	0.0094	0.9161
4	hsa-mir-3690-2	−1.9481	0.0105	0.9161
5	hsa-mir-3690-1	−1.9481	0.0105	0.9161
6	hsa-miR-483-3p	1.3561	0.0108	0.9161
7	hsa-miR-27a-5p	−1.3216	0.0110	0.9161
8	hsa-mir-579	1.1104	0.0136	0.9161
9	hsa-mir-133a-2	1.2010	0.0176	0.9161
10	hsa-miR-219a-1-3p	−1.4028	0.0188	0.9161
11	hsa-miR-133a-5p	1.4021	0.0191	0.9161
12	hsa-miR-493-3p	−1.3788	0.0195	0.9161
13	hsa-mir-133a-1	1.2001	0.0200	0.9161
14	hsa-miR-1245a	−1.3254	0.0214	0.9161
15	hsa-miR-23a-5p	−1.3435	0.0217	0.9161
16	hsa-miR-133a-3p	1.1719	0.0219	0.9161
17	hsa-mir-7704	−1.0055	0.0250	0.9161
18	hsa-miR-550a-3p	1.2909	0.0272	0.9161
19	hsa-miR-4508	−1.5594	0.0285	0.9161
20	hsa-mir-4508	−1.5594	0.0285	0.9161
21	hsa-miR-579-5p	1.2289	0.0339	0.9161
22	hsa-miR-215-5p	1.1979	0.0363	0.9161
23	hsa-miR-514a-3p	1.4606	0.0374	0.9161
24	hsa-mir-215	1.1756	0.0390	0.9161
25	hsa-mir-6818	−1.2591	0.0400	0.9161
26	hsa-mir-514a-3	1.4891	0.0413	0.9161
27	hsa-mir-514a-1	1.4891	0.0413	0.9161
28	hsa-mir-514a-2	1.4891	0.0413	0.9161
29	hsa-mir-3651	−1.6337	0.0423	0.9161
30	hsa-mir-1294	−1.0721	0.0426	0.9161
31	hsa-mir-135b	−1.6181	0.0428	0.9161
32	hsa-miR-135b-5p	−1.6110	0.0433	0.9161
33	SNORA3	−1.1678	0.0450	0.9161
34	hsa-miR-1294	−1.0974	0.0472	0.9161
35	hsa-mir-7854	−1.2553	0.0473	0.9161

## Supplementary Materials

The following are available online at http://www.mdpi.com/2073-4425/9/7/338/s1, Table S1: Characteristic of 18 genes enriched in Gene Ontologies (GO) in the category of biological process.

## References

[1] March L.M., Bachmeier C.J. (1997). Economics of osteoarthritis: A global perspective. Baillieres Clin. Rheumatol..

[2] White D.K., Master H. (2016). Patient-reported measures of physical function in knee osteoarthritis. Rheum. Dis. Clin..

[3] Jones G. (2016). What’s new in osteoarthritis pathogenesis?. Intern. Med. J..

[4] Hunter D.J., McDougall J.J., Keefe F.J. (2008). The symptoms of osteoarthritis and the genesis of pain. Rheum. Dis. Clin..

[5] Kidd B.L. (2006). Osteoarthritis and joint pain. Pain.

[6] Dieppe P.A., Lohmander L.S. (2005). Pathogenesis and management of pain in osteoarthritis. Lancet.

[7] Pincus T., Block J.A. (2009). Pain and radiographic damage in osteoarthritis. BMJ.

[8] Lee Y.C., Nassikas N.J., Clauw D.J. (2011). The role of the central nervous system in the generation and maintenance of chronic pain in rheumatoid arthritis, osteoarthritis and fibromyalgia. Arthritis Res. Ther..

[9] Schaible H.G., Richter F., Ebersberger A., Boettger M.K., Vanegas H., Natura G., Vazquez E., Segond von Banchet G. (2009). Joint pain. Exp. Brain Res..

[10] Wen Z.H., Chang Y.C., Jean Y.H. (2015). Excitatory amino acid glutamate: Role in peripheral nociceptive transduction and inflammation in experimental and clinical osteoarthritis. Osteoarthr. Cartil..

[11] Kidd B.L., Photiou A., Inglis J.J. (2004). The role of inflammatory mediators on nociception and pain in arthritis. Novartis Foundation Symposium.

[12] Sellam J., Berenbaum F. (2010). The role of synovitis in pathophysiology and clinical symptoms of osteoarthritis. Nat. Rev. Rheumatol..

[13] Klein K., Aeschlimann A., Jordan S., Gay R., Gay S., Sprott H. (2012). ATP induced brain-derived neurotrophic factor expression and release from osteoarthritis synovial fibroblasts is mediated by purinergic receptor p2x4. PLoS ONE.

[14] Bratus A., Aeschlimann A., Russo G., Sprott H. (2014). Candidate gene approach in genetic epidemiological studies of osteoarthritis-related pain. Pain.

[15] Kellgren J.H., Lawrence J.S. (1957). Radiological assessment of osteo-arthrosis. Ann. Rheum. Dis..

[16] Li B., Dewey C.N. (2011). RSEM: Accurate transcript quantification from RNA-Seq data with or without a reference genome. BMC Bioinform..

[17] Chen C.J., Servant N., Toedling J., Sarazin A., Marchais A., Duvernois-Berthet E., Cognat V., Colot V., Voinnet O., Heard E. (2012). ncPRO-seq: A tool for annotation and profiling of ncRNAs in sRNA-seq data. Bioinformatics.

[18] Griffiths-Jones S., Saini H.K., van Dongen S., Enright A.J. (2008). miRBase: tools for microRNA genomics. Nucleic Acids Res..

[19] Nawrocki E.P., Burge S.W., Bateman A., Daub J., Eberhardt R.Y., Eddy S.R., Floden E.W., Gardner P.P., Jones T.A., Tate J. (2015). Rfam 12.0: Updates to the RNA families database. Nucleic Acids Res..

[20] Robinson M.D., McCarthy D.J., Smyth G.K. (2010). Edger: A bioconductor package for differential expression analysis of digital gene expression data. Bioinformatics.

[21] Benjamini Y., Hochberg Y. (1995). Controlling the false discovery rate: A practical and powerful approach to multiple testing. J. R. Stat. Soc. Ser. B Stat. Methodol..

[22] Mi H., Poudel S., Muruganujan A., Casagrande J.T., Thomas P.D. (2016). Panther version 10: Expanded protein families and functions, and analysis tools. Nucleic Acids Res..

[23] Supek F., Bosnjak M., Skunca N., Smuc T. (2011). Revigo summarizes and visualizes long lists of gene ontology terms. PLoS ONE.

[24] Mootha V.K., Lindgren C.M., Eriksson K.F., Subramanian A., Sihag S., Lehar J., Puigserver P., Carlsson E., Ridderstrale M., Laurila E. (2003). PGC-1alpha-responsive genes involved in oxidative phosphorylation are coordinately downregulated in human diabetes. Nat. Genet..

[25] Subramanian A., Tamayo P., Mootha V.K., Mukherjee S., Ebert B.L., Gillette M.A., Paulovich A., Pomeroy S.L., Golub T.R., Lander E.S. (2005). Gene set enrichment analysis: A knowledge-based approach for interpreting genome-wide expression profiles. Proc. Natl. Acad. Sci. USA.

[26] Newman A.M., Liu C.L., Green M.R., Gentles A.J., Feng W., Xu Y., Hoang C.D., Diehn M., Alizadeh A.A. (2015). Robust enumeration of cell subsets from tissue expression profiles. Nat. Methods.

[27] Stephenson W., Donlin L.T., Butler A., Rozo C., Bracken B., Rashidfarrokhi A., Goodman S.M., Ivashkiv L.B., Bykerk V.P., Orange D.E. (2018). Single-cell RNA-seq of rheumatoid arthritis synovial tissue using low-cost microfluidic instrumentation. Nat. Commun..

[28] Hawker G.A., Mian S., Kendzerska T., French M. (2011). Measures of adult pain: Visual Analog Scale for Pain (VAS Pain), Numeric Rating Scale for Pain (NRS pain), Mcgill Pain Questionnaire (MPQ), Short-Form Mcgill Pain Questionnaire (SF-MPQ), Chronic Pain Grade Scale (CPGS), Short Form-36 Bodily Pain Scale (SF-36 BPS), and Measure of Intermittent and Constant Osteoarthritis Pain (ICOAP). Arthritis Care Res. (Hoboken).

[29] Karabis A., Nikolakopoulos S., Pandhi S., Papadimitropoulou K., Nixon R., Chaves R.L., Moore R.A. (2016). High correlation of VAS pain scores after 2 and 6 weeks of treatment with VAS pain scores at 12 weeks in randomised controlled trials in rheumatoid arthritis and osteoarthritis: Meta-analysis and implications. Arthritis Res. Ther..

[30] Descalzi G., Mitsi V., Purushothaman I., Gaspari S., Avrampou K., Loh Y.E., Shen L., Zachariou V. (2017). Neuropathic pain promotes adaptive changes in gene expression in brain networks involved in stress and depression. Sci. Signal..

[31] Lei J.X., Cassone C.G., Luebbert C., Liu Q.Y. (2011). A novel neuron-enriched protein SDIM1 is down regulated in Alzheimer’s brains and attenuates cell death induced by DNAJB4 over-expression in neuro-progenitor cells. Mol. Neurodegener..

[32] Cheng Y., Cawley N.X., Loh Y.P. (2014). Carboxypeptidase E (NF-α1): A new trophic factor in neuroprotection. Neurosci. Bull..

[33] Almeida R.D., Manadas B.J., Melo C.V., Gomes J.R., Mendes C.S., Graos M.M., Carvalho R.F., Carvalho A.P., Duarte C.B. (2005). Neuroprotection by BDNF against glutamate-induced apoptotic cell death is mediated by ERK and PI3-kinase pathways. Cell Death Differ..

[34] Johnson-Farley N.N., Patel K., Kim D., Cowen D.S. (2007). Interaction of FGF-2 with IGF-1 and BDNF in stimulating Akt, ERK, and neuronal survival in hippocampal cultures. Brain Res..

[35] Roux I., Safieddine S., Nouvian R., Grati M., Simmler M.C., Bahloul A., Perfettini I., Le Gall M., Rostaing P., Hamard G. (2006). Otoferlin, defective in a human deafness form, is essential for exocytosis at the auditory ribbon synapse. Cell.

[36] Wu W., Rahman M.N., Guo J., Roy N., Xue L., Cahill C.M., Zhang S., Jia Z. (2015). Function coupling of otoferlin with GAD65 acts to modulate GABAergic activity. J. Mol. Cell Biol..

[37] Cederquist G.Y., Luchniak A., Tischfield M.A., Peeva M., Song Y., Menezes M.P., Chan W.M., Andrews C., Chew S., Jamieson R.V. (2012). An inherited TUBB2B mutation alters a kinesin-binding site and causes polymicrogyria, CFEOM and axon dysinnervation. Hum. Mol. Genet..

[38] Lipina T.V., Prasad T., Yokomaku D., Luo L., Connor S.A., Kawabe H., Wang Y.T., Brose N., Roder J.C., Craig A.M. (2016). Cognitive deficits in Calsyntenin-2-deficient mice associated with reduced GABAergic transmission. Neuropsychopharmacology.

[39] Pannecoeck R., Serruys D., Benmeridja L., Delanghe J.R., van Geel N., Speeckaert R., Speeckaert M.M. (2015). Vascular adhesion protein-1: Role in human pathology and application as a biomarker. Crit. Rev. Clin. Lab. Sci..

[40] Stepp M.W., Folz R.J., Yu J., Zelko I.N. (2014). The c10orf10 gene product is a new link between oxidative stress and autophagy. Biochim. Biophys. Acta.

[41] Li X., Gibson G., Kim J.S., Kroin J., Xu S., van Wijnen A.J., Im H.J. (2011). MicroRNA-146a is linked to pain-related pathophysiology of osteoarthritis. Gene.

