# Supplementary material for: Pain-Associated Transcriptome Changes in Synovium of Knee Osteoarthritis Patients
Source: Genes (Basel). 2018 Jul 4;9(7):338. doi: 10.3390/genes9070338 (PMC6070955; doi:10.3390/genes9070338)
Supplement: Supplementary file 1 [file genes-09-00338-s001.pdf]

Table S1: Characteristic of 18 genes enriched in Gene Ontologies (GO) in the category of biological process

No.	Gene Symbol<br>Gene Name	PANTHER Family/Subfamily	PATHER Protein Class	log2 Ratio	pValue	Adjusted P value
**1**	**CPE**<br>Carboxypeptidase E	CARBOXYPEPTIDASE E (PTHR11532:SF59)	metalloprotease	1.107	2.13E-07	0.001172
**2**	**KSR1**<br>Kinase suppressor of Ras 1	KINASE SUPPRESSOR OF RAS 1 (PTHR23257:SF413)	non-receptor serine/threonine protein kinase<br>non-receptor tyrosine protein kinase	0.8648	9.15E-06	0.01909
**3**	**RASD1**<br>Dexamethasone-induced Ras-related protein 1	DEXAMETHASONE-INDUCED RAS-RELATED PROTEIN 1 (PTHR24070:SF255)	small GTPase	1.605	1.04E-05	0.01909
**4**	**ADCY3**<br>Adenylate cyclase type 3	ADENYLATE CYCLASE TYPE 3 (PTHR11920:SF291)	adenylate cyclase<br>guanylate cyclase	0.8997	1.29E-05	0.02012
**5**	**DUSP4**<br>Dual specificity protein phosphatase 4	DUAL SPECIFICITY PROTEIN PHOSPHATASE 4 (PTHR10159:SF111)	-	1.297	1.42E-05	0.02012
**6**	**PDE4D**<br>cAMP-specific 3',5'-cyclic phosphodiesterase 4D	CAMP-SPECIFIC 3',5'-CYCLIC PHOSPHODIESTERASE 4D (PTHR11347:SF91)	phosphodiesterase	1.083	1.47E-05	0.02012
**7**	**PLXNA4**<br>Plexin-A4	PLEXIN-A4 (PTHR22625:SF34)	tyrosine protein kinase receptor<br>signaling molecule<br>protein kinase	1.232	2.43E-05	0.02859
**8**	**CCL14**<br>C-C motif chemokine 14	C-C MOTIF CHEMOKINE 14 (PTHR12015:SF110)	chemokine	1.212	3.10E-05	0.03001

**9**	**DMD** Dystrophin	DYSTROPHIN (PTHR11915:SF261)	non-motor actin binding protein	0.9328	3.29E-05	0.03006
**10**	**AGT** Angiotensinogen	ANGIOTENSINOGEN (PTHR11461:SF13)	serine protease inhibitor	1.732	6.87E-05	0.05489
**11**	**SHC2** SHC-transforming protein 2	SHC-TRANSFORMING PROTEIN 2 (PTHR10337:SF5)	signaling molecule	1.574	7.00E-05	0.05489
**12**	**SFRP1** Secreted frizzled-related protein 1	SECRETED FRIZZLED-RELATED PROTEIN 1 (PTHR11309:SF87)	signaling molecule G-protein coupled receptor	0.953	7.41E-05	0.05551
**13**	**LMOD1** Leiomodin-1	LEIOMODIN-1 (PTHR10901:SF5)	non-motor actin binding protein	1.02	0.000143	0.09593
**14**	**CRISPLD2** Cysteine-rich secretory protein LCCL domain-containing 2	CYSTEINE-RICH SECRETORY PROTEIN LCCL DOMAIN-CONTAINING 2 (PTHR10334:SF64)	defense/immunity protein	1.631	0.000146	0.09593
**15**	**NTN1** Netrin-1	NETRIN-1 (PTHR10574:SF219)	receptor extracellular matrix linker protein	1.48	0.000154	0.09774
**16**	**IGF1** Insulin-like growth factor I	INSULIN-LIKE GROWTH FACTOR I (PTHR11454:SF4)	growth factor peptide hormone	1.159	0.000195	0.1009
**17**	**NTRK2** BDNF/NT-3 growth factors receptor	BDNF/NT-3 GROWTH FACTORS RECEPTOR (PTHR24416:SF136)	-	1.176	0.000195	0.1009
**18**	**PKDCC** Protein kinase domain-containing protein, cytoplasmic	PROTEIN KINASE DOMAIN-CONTAINING PROTEIN, CYTOPLASMIC (PTHR26392:SF77)	non-receptor serine/threonine protein kinase	0.9649	0.000196	0.1009
